# Angiogenesis in Inflammatory Bowel Disease

**DOI:** 10.3390/ph19071025

**Published:** 2026-06-30

**Authors:** Antoni Stadnicki, Anna Stadnicka, Wioletta Pollok-Waksmańska

**Affiliations:** 1 Faculty of Medicine, Jan Długosz University in Częstochowa, 42-200 Częstochowa, Poland; 2 Polonia University in Częstochowa, 42-200 Częstochowa, Poland; 3 District Hospital, 78-100 Kolobrzeg, Poland; astadnicka007@gmail.com; 4 CBT EDU Center, School of Cognitive-Behavioral Therapy in Szczecin, 70-712 Warsaw, Poland; 5 Faculty of Health Sciences, University of Bielsko-Biala, 43-309 Bielsko-Biala, Poland; wwaksmanska@ubb.edu.pl

**Keywords:** inflammatory bowel disease, IBD, angiogenesis, VEGF, intestinal barrier, microbiota, coagulation, bradykinin

## Abstract

The etiology of inflammatory bowel disease (IBD) is not precisely defined. However, it involves environmental factors, genetic predisposition, the involvement of gut microbiota, and abnormal immune response. Angiogenesis seems to be an integral part of IBD. Impairment of the intestinal barrier may represent an initiating or early feature of the disease. Disruption of the epithelial barrier leads to the translocation of microbiota and other antigens into the mucosa, resulting in an enhanced immune response, whereas damage to the vascular barrier is related to endothelial activation and pathologic angiogenesis, both of which promote inflammation. Angiogenesis during IBD is a very complex phenomenon that includes endothelial and immune cells, growth factors, cytokines, adhesion molecules, intestinal microbiota, and signal transduction. It seems that intestinal microvascular hemostasis shifts toward a prothrombotic state, and microthrombi formation exacerbates ischemia. The angiogenic process in IBD is regulated, at least in part, by the intestinal microbiota. Antiangiogenic therapy represents a novel and significant approach to the treatment of IBD. Biologic anti-inflammatory therapy for IBD simultaneously attenuates angiogenesis to a similar degree. However, the expression of VEGF and other growth factors may have dual and opposing effects, probably depending on the stage of the disease. Thus, anti-angiogenic treatment in patients with IBD remains controversial, and clinical trials of anti-angiogenic agents are warranted.

## 1. Inflammatory Bowel Disease

Crohn’s disease and ulcerative colitis (UC) are the two principal forms of inflammatory bowel disease (IBD), characterized by chronic intestinal and systemic inflammation and a relapsing-remitting course. The etiology and pathogenesis of IBD are still not fully understood. However, they involve genetic and environmental factors and are associated with immunologic alterations driven, at least in part, by the intestinal microbiome [1].

IBD has classically been considered a disease of Western countries, but over the past few decades, its incidence has increased rapidly in newly industrialized regions of the world. UC primarily affects the large bowel, whereas Crohn’s disease may affect any part of the gastrointestinal tract, from the oral cavity to the rectum. Approximately 20–30% of IBD cases are associated with extraintestinal complications [1].

The intestinal barrier consists of epithelial and vascular layers that protect the host from potentially harmful components. When the intestinal barrier is disrupted, bacterial and other antigens may enter the usually sterile submucosa and alter the immune response, thereby contributing to the pathogenesis of IBD. Under normal conditions, immunologic mechanisms suppress inflammation. However, in genetically susceptible individuals, the inflammatory response is altered and amplified as a result of defective innate immune-cell function and regulation, impaired epithelial barrier function, and pathologic angiogenesis. Extensive tissue injury is characterized by increased expression of adhesion molecules in the vessel wall, inflammatory cell infiltration, and the release of cytokines and other inflammatory mediators. Intestinal inflammation in patients with IBD may also promote compositional and metabolic alterations in commensal microbiota, a process referred to as dysbiosis [2]. As discussed below, decreased microbial diversity, especially among members within the Firmicutes and Bacteroidetes phyla, results in a reduction of butyrate production by these commensal microorganisms [2]. These alterations contribute to impairment of the protective effects of the intestinal barrier function and to the perpetuation of chronic inflammation in IBD.

In recent years, many reports have presented a role of intestinal microbiota in intestinal homeostasis and intestinal barrier dysfunction in IBD. In light of current evidence, this review focuses primarily on proangiogenic activators, the contribution of microbiota to the activation of the intestinal microvasculature, the relationship between intestinal barrier dysregulation and pathologic angiogenesis, and the effectiveness of anti-angiogenic therapy in IBD.

## 2. Angiogenesis

Angiogenesis, defined as the process of new vessel formation, physiologically takes place during embryonic development and the menstrual cycle. However, under pathological conditions, the formation of new vessels may persist, which can induce or exacerbate several pathological conditions, such as cancer and chronic inflammation. Angiogenesis consists of multiple steps, including the stimulation and proliferation of vascular endothelial cells (ECs), maturation and lumen formation, degradation of the basement membrane and extracellular matrix (ECM), and vascular remodeling [3]. Pathological angiogenesis is a component of inflammation and is perpetuated by the immune response. Chronic inflammation and angiogenesis play a significant role in the pathogenesis of various chronic diseases, such as rheumatoid arthritis, psoriasis, and metabolic syndrome [4]. Evidence has accumulated indicating that pathological angiogenesis is pivotal in IBD (Table 1) [5].

### 2.1. Activators of Angiogenesis

There are multiple components of the angiogenic cascade. However, vascular endothelial growth factor (VEGF) is the principal angiogenic factor. Members of the VEGF family bind to three specific receptors (VEGFR1, VEGFR2, and VEGFR3). VEGF-A acts through four main isoforms; VEGF-A165 is the major activator of pathologic angiogenesis and acts by binding to VEGFR2 [6]. VEGF mediates angiogenesis by stimulating EC proliferation and migration and, in addition, by inhibiting EC apoptosis [7]. VEGF may also increase vascular permeability and activate metalloproteinases (MMPs), which participate in the degradation of extracellular matrix (ECM) [8].

Angiogenesis during chronic inflammation is considered to be induced primarily by hypoxia [9,10], with hypoxia-inducible factors-1 and -2 (HIF-1 and HIF-2) transcriptionally activating the expression of VEGF-A [11]. VEGF activates nuclear factor *κ*B (NF-*κ*B) and, in turn, stimulates the release of pro-inflammatory cytokines, such as IL-1, TNFα, growth factors and chemokines from leukocytes and ECs [12,13,14,15].

Growth factors have been implicated in the activation and maintenance of angiogenesis. Among these growth factors, fibroblast growth factor (FGF), transforming growth factor-β (TGF-β) and platelet-derived growth factor (PDGF) play particularly important roles. PDGF is released in response to hypoxia, cytokines, and other growth factors, and thrombin. It mediates the recruitment of vascular muscle cells to the angiogenic milieu [16]. FGF supports angiogenesis through EC proliferation, differentiation, and migration, whereas TGF-β modulates EC survival and differentiation and regulates vascular homeostasis [17,18]. In addition, hepatocyte growth factor (HGF) mediates the proliferation and differentiation of ECs [19], and placental growth factor (PGF) is thought to be a sensitive promoter of pathologic angiogenesis [20].

Besides classical angiogenic factors, the angiopoietin/tyrosine-kinase receptor (Ang/Tie) system plays a significant role in the late phase of angiogenesis through the maturation and stabilization of blood vessels [21,22]. Angiopoietins may regulate angiogenesis through the Tie-2 receptor. Activated ECs produce the proangiogenic mediator angiopoietin-2, which is not typically expressed in normal vessels. Angiopoietin-1 acts as a regulator of blood vessel maturation and has anti-inflammatory properties, whereas Angiopoietin-2 facilitates EC activation in response to VEGF-A and other classical growth factors. It promotes angiogenesis by increasing EC sensitivity to VEGF-A-mediated proliferation [23,24]. In fact, coordination of Ang-Tie-2 signaling and NF-*κ*B activation may contribute to the vicious circle of inflammation and angiogenesis [25,26].

### 2.2. Antiangiogenic Factors

Thrombospondins (TSPs), calcium-binding extracellular glycoproteins, are well-known antiangiogenic factors. They inhibit angiogenesis by stimulating EC apoptosis and regulating inflammation [27,28]. TSP-1, the major antiangiogenic molecule, has been shown to be upregulated by HIF-1 and to enhance phagocytosis by macrophages and neutrophils under hypoxic conditions [29]. Other factors that inhibit angiogenesis include angiostatin and endostatin, which are 20 kDa and 50 kDa fragments derived from plasminogen and collagen XVIII, respectively. These molecules may induce EC apoptosis and inhibit EC proliferation and migration.

## 3. Intestinal Barrier

Epithelial cells, enterocytes and mucus-producing goblet cells, and the Paneth cells constitute the first layer that isolates the microorganisms in the gut microenvironment [30]. This intestinal epithelial barrier prevents the penetration of microbiota or microbial products into the tissue. Directly below the epithelial barrier, the vascular barrier has been identified. It consists of ECs, a continuous basement membrane, and a regular ECM. This is the second protective layer, which prevents microbial penetration into the vasculature [31,32]. At present, there is agreement that disruption of the intestinal barrier, accompanied by bacterial dissemination, probably represents an initiating or early feature of the disease [33,34].

Mediators of inflammation, including cytokines such as IFN-γ, IL-6, and TNF-α, have been found to increase intestinal permeability in experimental colitis models and IBD [35]. On the other hand, recent reports in patients with IBD have indicated that increased epithelial permeability may precede exacerbation of bowel inflammation, suggesting the causal significance of epithelial barrier disruption in intestinal inflammation [35]. The cytokine cascade may also activate ECs, including the expression of cell adhesion molecules (CAMs), such as E-selectin, intercellular adhesion molecule 1 (ICAM-1), vascular cell adhesion molecule 1 (VCAM-1), and platelet endothelial cell adhesion molecule 1 (PECAM-1, also known as CD31), and the synthesis of chemokines, thereby promoting leukocyte infiltration and mucosal damage [36].

At the molecular level, the mitogen-activated protein kinase (MAPK) signaling pathway has been demonstrated to play a significant role in the regulation of CAMs and the generation of chemokines by activated human intestinal microvascular endothelial cells (HIMECs), as well as in lymphocyte extravasation [37]. In the inflamed mucosa of patients with IBD, increased levels of phosphorylated and, therefore, activated MAPK have been detected in the microvasculature [37].

VEGF, which is synthesized by the intestinal epithelium, may provoke loosening of tight junctions (TJs) and adherens junctions (AJs) between enterocytes and impair the intestinal barrier, causing the translocation of intestinal bacteria from the intestinal lumen, leading to an enhanced immune response in genetically predisposed hosts [38]. Permeability in the intestinal microvasculature is controlled by TJs and AJs, as it is in the epithelial barrier [39]. AJs are formed by vascular endothelial cadherin (VE-cadherin) and β-catenin [40], whereas TJs in the intestinal endothelium are composed mainly of junctional adhesion molecule (JAM)-A, occludin, and cingulin [31]. In addition, members of the claudin family, including claudin-3, claudin-5, and claudin-12, together with enteric glial cells associated with the intestinal endothelium [41,42], contribute to the development of the gut-vascular barrier, which regulates soluble paracellular transport in a manner comparable to that of the brain–blood barrier (BBB) [43,44]. However, in contrast to the BBB, which has a size-exclusion threshold of 500 Da, the gut–vascular barrier is permeable to molecules as large as 4 kDa [31]. Thus, intestinal permeability is mediated by disturbances in both the epithelial and endothelial barriers.

## 4. Intestinal Hemostasis in IBD

Ischemia-associated inflammation in the intestinal microvasculature, resulting from pro-coagulant activity and the formation of thrombi, further enhances tissue damage and pathologic angiogenesis. Several decades ago, Wakefield et al. [45,46] observed granulomatous vasculitis in the intestinal microvasculature during the early stage of Crohn’s disease, which suggests its pathogenic significance. Capillary thrombi associated with fibrin deposition and the expression of tissue factor were also observed in intestinal vasculature of patients with Crohn’s disease and UC [47,48].

Activated platelets detected in the intestinal microcirculation of patients with IBD may activate endothelial cells through the expression of CD40. Danese et al. [49] demonstrated increased platelet expression of surface CD40 ligand (CD40L) and elevated plasma concentrations of platelet-derived soluble CD40L in patients with UC and Crohn’s disease compared with healthy subjects. They also observed CD40L-positive platelets adherents to ECs in the intestinal circulation, which may initiate an inflammatory response [50].

In addition, the interaction between platelet-expressed CD40L with CD40 expressed on vascular cells may increase the expression of inflammatory cell adhesion molecules, including ICAM-1 and VCAM-1, and promote leukocyte migration into the extravascular space. Independently, platelet adhesion to inflamed intestinal endothelial cells may favor angiogenesis through the release of VEGF and platelet-derived growth factor (PDGF), which has also been proposed as a potential therapeutic target in IBD [51].

In the inflamed mucosa of patients with IBD, de Jong et al. [52] reported decreased tissue plasminogen activator (t-PA) expression and increased urokinase plasminogen activator (u-PA). Unlike t-PA, u-PA is less fibrin-dependent; thus, plasmin generated through u-PA activity may act as a proinflammatory protease. The cytokines IL-1 and TNF–α are able to exert procoagulant effects by inducing tissue factor expression in ECs, platelets and monocytes and to suppress the anticoagulant potential of thrombomodulin through downregulation of the endothelial protein C receptor, which is normally maintained by ECs [53]. This disruption of the protein C system is also related to increased adherence of ECs, thereby supporting leukocyte recruitment. Recent investigations have indicated that the protein C pathway is expressed not only in ECs of the intestinal vessels but also in epithelial cells, and that it plays a significant role in enhancing the integrity of tight junctions [54]. In the intestinal epithelium of patients with Crohn’s disease and UC, protein C expression is altered, which may increase intestinal permeability [55].

## 5. Angiogenesis in IBD

Vascular quality is probably critical to the pathogenesis of IBD (Table 2). In fact, newly formed vessels in IBD tissues are highly disorganized and leaky, as indicated by associated tissue edema [56,57]. The induction and propagation of angiogenesis during IBD have been partly connected with hypoxia. In fact, in the IBD mucosa, hypoxia-inducible factors-1 and -2 transcriptionally activate the expression of VEGF-A [11]. The VEGF gene may also be activated by other factors of significance in IBD, such as growth factors, mainly EGF and TGF-β, and cytokines, including TNFα, IL6 and Il-1β [8,58]. We and others have found increased VEGF expression in both endothelial cells and epithelial cells in the inflamed intestine of patients with IBD [5,59]. Increased VEGF levels in both serum and plasma have been observed in patients with IBD, which may reflect VEGF overexpression in inflamed intestinal tissue [56,59,60,61]. In addition, increased VEGF levels have been found in mucosal extracts obtained from patients with IBD compared with controls [5].

**Figure 1:**
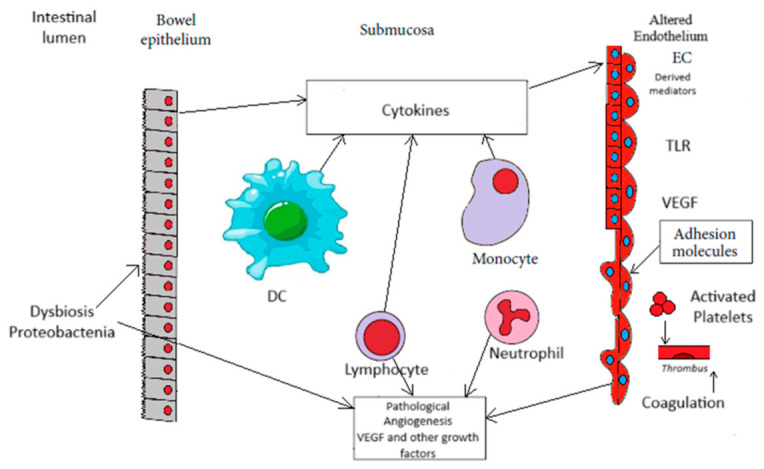
Main factors driving angiogenesis in inflammatory bowel disease. Schematic representation of the bowel lumen, inflamed intestinal mucosa/submucosa, and activated and altered endothelium. Infiltrating neutrophils (N), monocytes/macrophages (M), lymphocytes (L), and dendritic cells (DCs) secrete cytokines, growth factors, and other factors that stimulate endothelial cells (ECs). Activated ECs produce vascular endothelial growth factor (VEGF) and express Toll-like receptors (TLRs), which are specific receptors for bacterial products. Activated ECs expressing adhesion molecules promote leukocyte recruitment to the mucosa/submucosa and platelet adhesion, whereas activation of coagulation and platelets causes thrombus formation in the microvasculature and, in turn, ischemia. Vascular dysfunction leads to neovascularization and vascular remodeling, which characterize pathologic angiogenesis.

**Figure 2:**
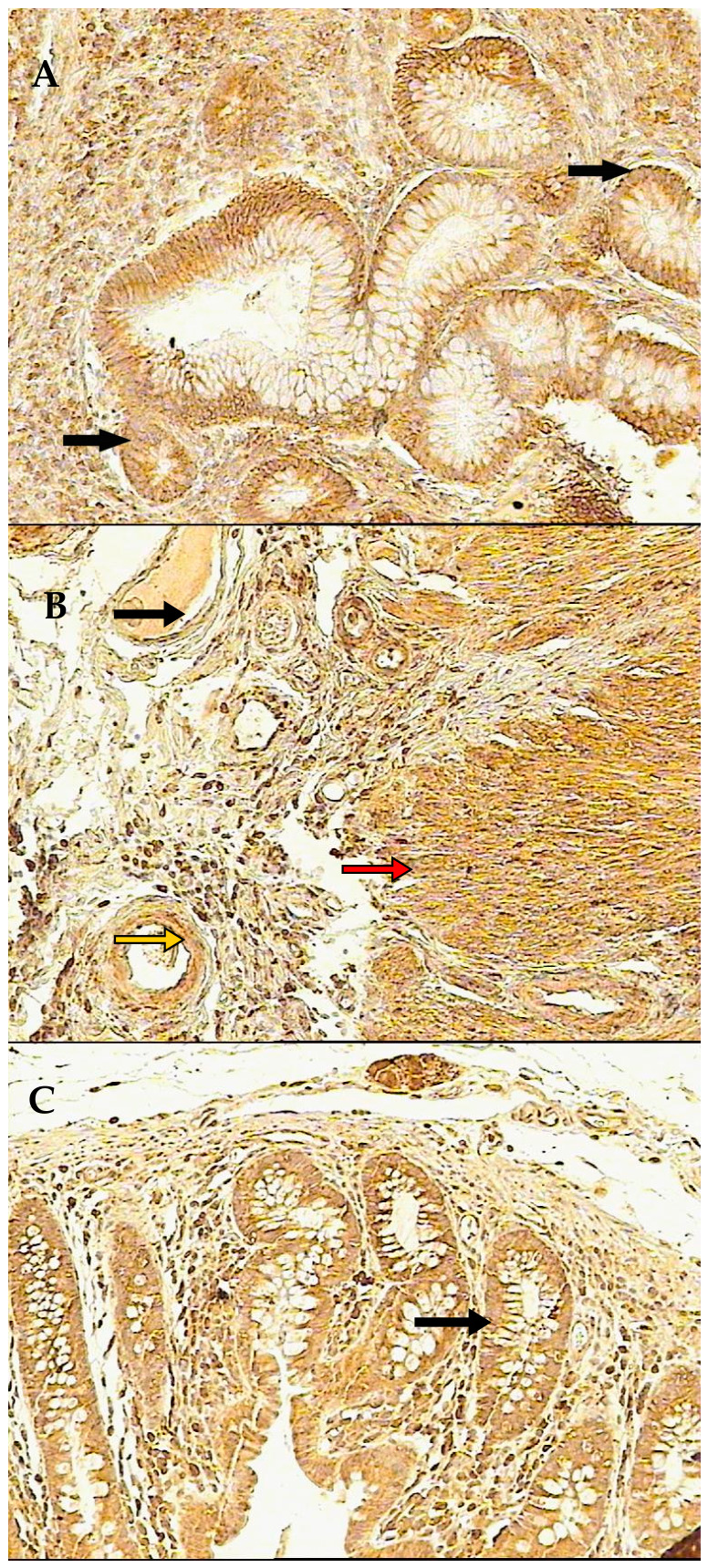
VEGF immunohistochemical localization in UC-inflamed intestinal tissue and normal colon (×100). (**A**) Colon in the active phase of UC; arrows indicate specific staining within enterocytes. (**B**) Colon in the active phase of UC; yellow arrow, specific staining in vascular endothelium; black arrow, specific staining in the vessel lumen; red arrow, specific staining within intestinal smooth muscle cells. (**C**) Normal colon; arrow indicates specific staining in enterocytes (from Ref. [60], with permission of Elsevier Publishing).

**Figure 3:**
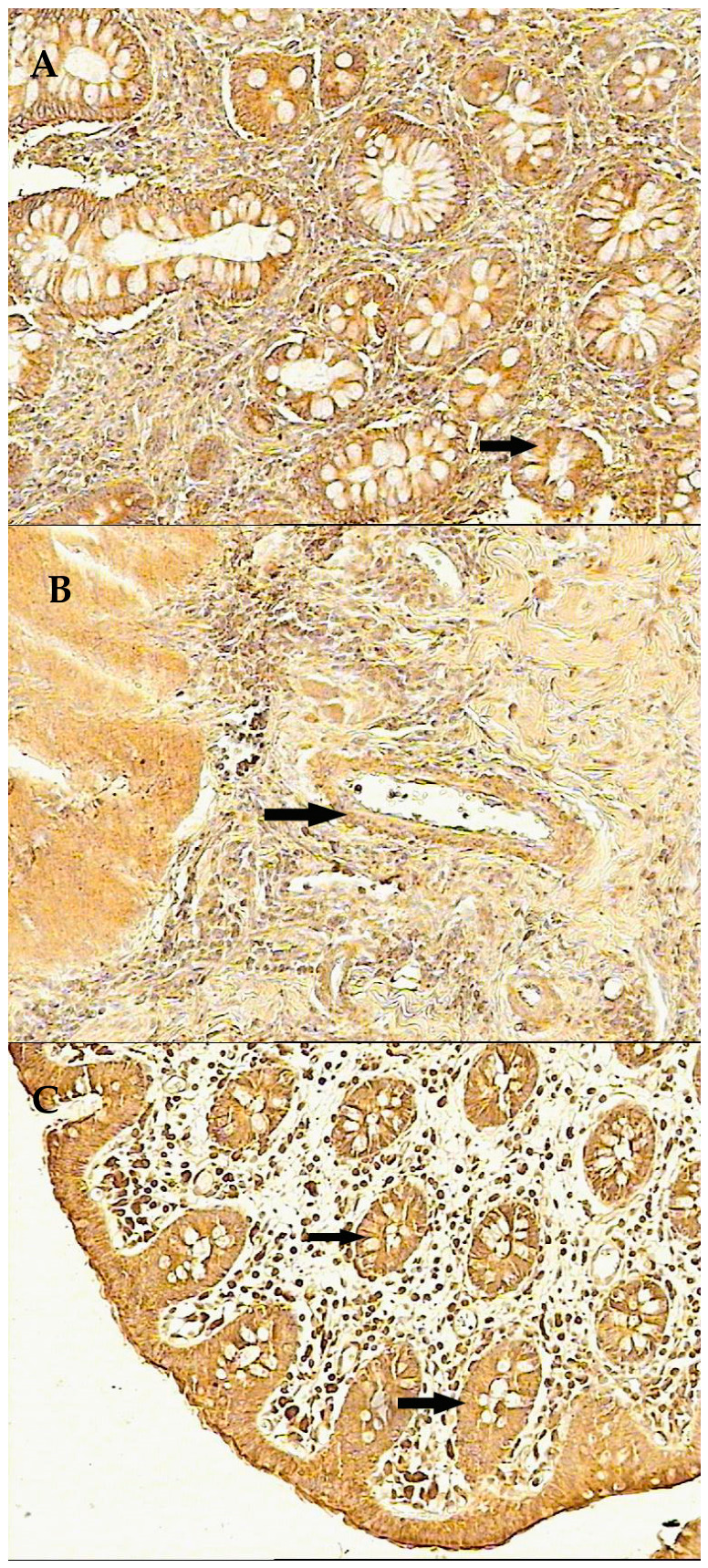
Flt-1 receptor localization in UC-inflamed intestinal tissue and normal colon (×100). (**A**) Colon in the active phase of UC; arrow indicates specific staining in enterocytes. (**B**) Colon in the active phase of UC; arrow indicates specific staining in the endothelium. (**C**)Normal colon; arrows indicate specific staining in enterocytes (from [59], with permission of Elsevier Publishing).

Danese et al. [5] were the first to document the significance of angiogenesis as a novel component of the pathogenesis of both UC and Crohn’s disease (Figure 1). These authors demonstrated a higher density of microvessels, as well as increased VEGF expression, in the intestinal mucosa during both active and inactive phases of UC and Crohn’s disease, compared with controls. It appears that VEGF may activate two tyrosine kinase receptors: Flt-1 (fms-like tyrosine kinase-1 receptor) and KDR (kinase domain receptor) [38,62]. The Flt-1 receptor has a higher affinity for VEGF than the KDR receptor. We observed significant increases in VEGF and Flt-1 gene expression, as well as higher levels of VEGF and Flt-1 protein, in the intestinal mucosa during active UC compared with the control group and the inactive UC phase. However, we found only trace KDR gene expression in the active UC phase [59]. Previously, German investigators, Griga et al. [63,64], reported stronger immunohistochemical staining for VEGF protein in the epithelium and lamina propria of colonic tissue in active UC than in inactive UC and normal colonic tissue, which is in line with our results (Figure 2 and Figure 3). In sharp contrast, Greek investigators, Giatromanolaki et al. [11] and Kapsoritakis et al. [65], using immunohistochemical methods, observed only weak VEGF-specific staining in endothelial cells and enterocytes in the inflamed colon of patients with UC. One could postulate that geographical differences, such as those between Northern Europe and the Mediterranean region, together with environmental factors and/or unknown genetic patterns, may modulate intestinal levels of VEGF and its receptors.

Endothelial damage may precede epithelial barrier dysfunction in IBD. Investigators at La Jolla University, using functional, morphologic, and molecular biologic approaches in four animal models of UC, indicated that endothelial permeability occurs earlier than epithelial barrier dysfunction and is followed by erosions, ulceration, and inflammation, suggesting that endothelial disruption might be critical in disease pathogenesis [66]. In addition, Lakatos et al. [67] showed that serum concentrations of MMP-9 and the tissue inhibitors of metalloproteinases TIMP-1 and TIMP-2 were higher in patients with UC and Crohn’s disease than in controls, and that MMP-9 and TIMP levels correlated significantly with disease activity. Thus, MMPs may contribute to tissue-remodeling processes in IBD, and serum MMP-9 and TIMP might be used as biomarkers of disease activity.

Elevated levels of other angiogenic growth factors, including basic fibroblast growth factor (bFGF) and platelet-derived growth factor (PDGF), have been found in the inflamed mucosa and blood of patients with IBD [57,68,69,70]. Placental growth factor (PlGF), a specific regulator of pathological angiogenesis, was found to be increased in the serum of patients with IBD [20,71]. Recently, Zhou et al. demonstrated the proangiogenic effects of PlGF on human intestinal microvascular endothelial cells (HIMECs) through activation of the PI3K/Akt signaling pathway [20].

With respect to the angiopoietin system, increased serum levels of Ang-2 and Tie-2 have been observed in patients with IBD [72]. Subsequent studies demonstrated higher immunoreactivity for Ang-1 and Ang-2 in the epithelium of crypt abscesses from patients with active UC compared with patients with UC in remission [73]. These findings suggest that the angiogenic response is regulated by the Ang/Tie system and that this pathway may play a role in the progression of UC. Another distinctive molecular feature of ECs within angiogenic vessels is the expression of the adhesion molecules integrins αvβ3 and αvβ5, which are involved in T-cell recruitment to the intestinal mucosa. Importantly, increased αvβ3 expression has been shown in the mucosa of patients with IBD [5,74].

In addition, kinins have been suggested to participate in angiogenesis. Kinins may mediate angiogenesis through upregulation of basic FGF via the B1 bradykinin receptor and through activation of VEGF synthesis via both B1 and B2 bradykinin receptors [75]. Our data [76] demonstrated the presence of B1 and B2 receptors in intestinal tissues from patients with IBD and indicated that B1 receptor upregulation is critical for kinin function. Kinins can stimulate cellular proliferation and interact with growth factors. Bradykinin, acting as a growth factor and as a member of the autacoid family, contributes to increased capillary permeability and edema. Thus, kinins most likely support angiogenesis in IBD, although the relationship between kinins, cytokines, and growth factors in this context requires further investigation.

Chloride channels (ClCs), which are expressed in intestinal mast cells and epithelial cells, support tight-junction integrity and regulate chloride ion transport, thereby contributing to intestinal barrier function. The recent report indicates that dysregulation of these channels in mast cells may mediate mast-cell activation and degranulation, as well as immune-cell recruitment in inflamed tissue, leading to barrier dysfunction and aggravation of certain IBD symptoms, particularly diarrhea [77]. Importantly, the number of mast cells and the expression of mast-cell tryptase, a marker of mast-cell degranulation, have been found to be increased in the colonic mucosa and submucosa in both experimental models of IBD and human IBD [78]. Thus, chloride channel modulation in mast cells may have therapeutic potential in IBD.

### 5.1. Angiogenesis and Microbiome in IBD

The healthy intestinal microbiota consists predominantly of members of the phyla *Firmicutes*, *Bacteroidetes*, *Actinobacteria*, and *Proteobacteria*, with *Firmicutes* and *Bacteroidetes* being dominant [79]. Intestinal tissue injury and disruption of the intestinal barrier lead to dysregulation of the intestinal milieu and altered interactions between mucosal components and the commensal microbiota. This disequilibrium affects the function of the barrier and, conversely, results in alterations in the composition of the commensal microbiota, leading to dysbiosis [2,80]. Recent reports have indicated that alterations in the gut microbiome in IBD are characterized by a less diverse gut microbial composition, resulting from a decrease in commensal anaerobic bacteria, particularly members of the *Firmicutes* and *Bacteroidetes* phyla, and an increase in the abundance of Gram-negative *Proteobacteria* [81].

It is evident that intestinal vascular remodeling and renewal are disturbed in dysbiosis, particularly under conditions of intestinal inflammation. However, the effects of individual components of the gut microbiota observed in in vitro and experimental models remain unclear. The impact of pathogenic *Escherichia coli* on pathological angiogenesis has been shown to involve the induction of HIF-1α overexpression and upregulation of IL-8, VEGF, and Twist1 gene expression, with simultaneous downregulation of E-cadherin expression [82]. *Bacteroides fragilis* toxin (BFT), secreted by enterotoxigenic *Bacteroides fragilis* (ETBF), induces degradation of E-cadherin protein in intestinal epithelial cells. This process activates the β-catenin-TCF nuclear signaling pathway, which in turn stimulates the production of interleukin-8, thereby promoting inflammatory responses and angiogenesis [83]. *Clostridium perfringens* produces toxins that may influence angiogenesis. For example, *Clostridium perfringens* type C produces beta-toxin, which binds to intestinal endothelial cells and forms oligomeric pores on the cell surface, thereby disrupting intestinal barrier function [84].

Dysbiosis in IBD is characterized not only by alterations in microbial composition but also by metabolic derangements, mainly decreased production of short-chain fatty acids (SCFAs), increased hydrogen sulfide (H_2_S) production, and altered bile acid and tryptophan metabolism, which lead to impaired epithelial homeostasis and promote inflammation.

In fact, increasing the concentrations of SCFAs within the intestinal lumen through ingestion of prebiotics, defined as oligosaccharides associated with plant-derived fiber, promotes the growth of protective bacteria and limits the contribution of Gram-negative bacteria [85]. SCFAs have been shown to restore mucosal barrier function, increase mucus production, stimulate the synthesis of immunosuppressive cytokines, such as IL-10, and decrease the generation of pro-inflammatory mediators. SCFAs may also increase the production of immunosuppressive regulatory T cells (Tregs) that express the transcription factor Foxp3, a key regulator of intestinal inflammation [86].

Interestingly, the relationship between the gut–vascular barrier and the microbiota has been shown to enhance angiogenesis, as observed in human IBD and murine models of colitis [41,87,88]. In fact, intestinal microbes are a source of angiogenic activators in the form of ligands for Toll-like receptors (TLRs) and protease-activated receptors (PARs) [87,88].

Ex vivo activated intestinal vessels, as well as human intestinal microvascular endothelial cells (HIMECs) exposed to microbial products, may undergo angiogenic sprouting. These effects are mediated through Toll-like and NOD-like receptors (TLRs and NLRs), which are components of innate immune responses [89]. Expression of TLRs on vascular endothelial cells is upregulated by vascular inflammation as well as by bacterial lipopolysaccharide (LPS).

Recently, it has been shown that activation of TLRs and NLRs by specific bacterial ligands selectively upregulates the levels of carcinoembryonic antigen-related cell adhesion molecule 1 (CEACAM1) produced by HIMECs and simultaneously induces angiogenesis. These results suggest that cooperation between endogenous and exogenous innate immune factors, mediated by the reciprocal interaction between CEACAM1 and the microbiota, is essential for the promotion of intestinal angiogenesis in IBD [90].

Microbial biomarkers are emerging as promising noninvasive tools to predict disease activity, disease risk, and recurrence after surgery. The most cost-effective and a less invasive method is the analysis of bacterial DNA in plasma. Importantly, bacterial genomic fragments (bactDNA) have been detected in the blood of up to 50% of patients with IBD [91], and bacterial DNA translocation can be regarded as a risk factor for relapse within 6 months in patients with Crohn’s disease; it is also independently associated with an increased risk of hospitalization and the need for corticosteroid therapy [92]. Recent reports indicate that a combination of sequencing techniques, including methylation-based human cell-specific profiling, together with shotgun metagenomics, may be used to characterize the human and microbial DNA content in feces. Combining neutrophil and other cell-type fecal DNA fractions has been demonstrated to provide a noninvasive approach for distinguishing between inactive and active IBD and may improve disease monitoring [93]. Another approach that may identify potential targets to support diagnosis and guide therapeutic strategies in IBD involves stool 16S rRNA gene sequencing and multisystem metabolomic phenotyping using nuclear magnetic resonance and mass spectrometry, followed by integrative network analysis to delineate novel microbiota–metabolome interactions [94].

In addition, some authors have reported endotoxemia and LPS-binding protein in the systemic circulation of patients with IBD [95,96]. Of note, a link exists between the gut–vascular barrier and blood–brain barrier (BBB) dysfunction in IBD. Depression and anxiety disorders have been demonstrated in up to 40% of patients with active IBD [97], and cognitive impairment has been reported less frequently [98,99]. Thus, circulating bacterial products and pro-inflammatory mediators in patients with IBD may contribute to central nervous system dysfunction.

It is documented that, in IBD, the microbiota and its products may modulate the function of immune cells as well as nonimmune cells, primarily ECs, and thus may play a role in the initiation or progression of inflammation. Importantly, in IBD, microbial products may trigger angiogenesis by activating TLRs in ECs. Thus, it is tempting to suggest that a reduction in gut microbial load might suppress angiogenesis.

### 5.2. Treatment of Angiogenesis in IBD

TNF-α, as a central mediator of inflammatory responses, has been documented in both Crohn’s disease and UC. Thus, anti-TNF-α agents are currently used as biologic therapy for moderate-to-severe active Crohn’s disease and UC [100]. In a clinical study, Italian investigators demonstrated the ability of infliximab to affect mucosal angiogenesis in patients with Crohn’s disease, showing that infliximab administration downregulates mucosal angiogenesis. This finding suggests that inflammation-driven angiogenesis in the gut mucosa may contribute to the therapeutic efficacy of TNF-α blockade [101]. Importantly, Algaba et al. showed that circulating VEGF and angiopoietin-1 levels decreased during anti-TNF therapy in patients with UC and Crohn’s disease [102]. Whether these changes represent signs of clinical improvement remains a matter of debate. In addition, treatment with infliximab downregulates the CD40/CD40L pathway in patients with Crohn’s disease [103], which may improve endothelial dysfunction by reducing thrombus formation in the intestinal vasculature.

Therapeutic strategies targeting VEGF represent an interesting approach to reducing intestinal angiogenesis and mucosal inflammation. Upregulation of VEGF, a potent vascular permeability factor, during the early stages of UC may provide evidence of impaired endothelial barrier function. Tolstanova et al. [104] showed, in an experimental rat model of UC, that an anti-VEGF antibody reduced colonic vascular permeability and improved morphologic signs of colitis, indicating the pathogenic role of VEGF. Other proangiogenic growth factors, such as placental growth factor (PlGF), are upregulated in patients with IBD through activation of the tyrosine kinase–phosphoinositide 3-kinase/protein kinase B (PI3K/Akt) signaling pathway. Thus, PlGF/PI3K/Akt signaling may represent an appropriate therapeutic target in IBD [20].

Bevacizumab, a humanized IgG1 monoclonal antibody directed against VEGF-A, is used as an antiangiogenic agent in various cancers, including colorectal cancer. However, to date, there are only case reports showing the beneficial effects of bevacizumab (an anti-VEGF antibody) and sunitinib (a multiple tyrosine kinase inhibitor targeting VEGF and PDGF signaling) in patients with Crohn’s disease [105,106]. Importantly, adverse effects of bevacizumab may include impaired wound healing, bleeding, and even intestinal perforation, although such complications are rare [107,108]. Moreover, in light of the case report by Loriot et al. [109], antiangiogenic treatment may exacerbate IBD. Recently, the safety of bevacizumab in association with chemotherapy in colorectal cancer coexisting with IBD has been retrospectively evaluated. The authors documented that bevacizumab is safe in cancer patients with IBD, and no clinical IBD exacerbations were found during bevacizumab treatment [110].

Another option for vessel-directed therapy in IBD involves anti-adhesion molecules, such as vedolizumab and etrolizumab [111]. Vedolizumab, an anti-α4β7 integrin-specific antibody, may induce long-term remission in refractory patients with Crohn’s disease and UC [112,113]. This drug blocks the interaction between α4β7 and mucosal addressin cell adhesion molecule 1 (MAdCAM-1), which is involved in leukocyte recruitment, whereas etrolizumab, a monoclonal antibody against MAdCAM-1, acts as a target for the β7 subunit and suppresses the accumulation of T lymphocytes [114]. Of note, Danese et al., in an experimental mouse model of ulcerative colitis, demonstrated inhibition of angiogenesis using the integrin αvβ3 inhibitor ATN161, with a concomitant decrease in intestinal inflammatory changes [115]. However, it should be emphasized that the beneficial effects of antiangiogenic drugs as adjunctive IBD therapy remain controversial. In fact, to date, no clinical trial has been performed using antiangiogenic therapy in patients with IBD.

Among possible microbiota-targeted interventions, probiotic administration has shown limited effectiveness, in part because of the limited number of microbial species currently available as probiotics. In contrast, fecal microbiota transplantation (FMT) replaces the intestinal milieu of the recipient with a fecal solution from a donor [116]. Successful results from the use of FMT for recurrent *Clostridioides difficile* infection have indicated new therapeutic approaches for IBD, aimed at replacing the existing microbiota and increasing the abundance and metabolic activity of protective bacteria. A number of clinical trials in patients with UC and Crohn’s disease have demonstrated the safety of FMT but variable efficacy in achieving clinical remission and maintaining remission after remission has been achieved with pharmacologic therapy [117]. The involvement of angiogenesis in the efficacy of FMT has been demonstrated in a murine irradiation model. In this model, FMT alleviated and protected against radiation-induced intestinal injury through upregulation of intestinal VEGF expression [118]. Expanding insights into donor microbial composition, recipient factors, and post-transplant microbial profiles may help determine the optimal frequency of FMT administration and predict successful FMT outcomes.

### 5.3. Dual Role of Angiogenesis

The data from numerous studies indicate the importance of angiogenesis in IBD, as it may initiate and/or amplify chronic inflammation and fibrosis in the intestine. VEGF induces abnormal angiogenesis, and other growth factors, mainly placental growth factor (PlGF), basic fibroblast growth factor (bFGF), and platelet-derived growth factor (PDGF), may play a critical role in the pathogenesis of IBD [60].

The function of angiogenesis in IBD pathogenesis remains poorly understood because of its opposing effects. On the one hand, angiogenesis may initiate and perpetuate inflammation by stimulating the influx of inflammatory mediators and cells. On the other hand, angiogenesis is necessary to supply oxygen and nutrients to promote healing of the damaged mucosa. Despite the activation of angiogenic signals, impaired mucosal healing remains a major feature of IBD. Thus, it is important to identify the inducers and modulators of pathological angiogenesis. PlGF has been identified as a marker of pathological angiogenesis and plays a critical role only in abnormal angiogenesis. Interestingly, inhibition of PlGF did not affect quiescent vessels in healthy organs [119].

On the other hand, these growth factors can be favorable in healing and repair processes [120]. It has been shown in experimental studies that administration of PDGF or FGF significantly accelerated healing in UC [121]. In fact, EGF enemas have been demonstrated to be useful in patients with UC [122]. The researchers at La Jolla University documented in rat models that the molecular mechanisms underlying the healing effects of bFGF in UC are associated with increased cell proliferation, particularly angiogenesis, and a simultaneous decrease in cytokine levels and inflammatory-cell infiltration [123]. Other authors also demonstrated that activation of Rac1, a key member of the Rho guanosine triphosphatase (GTPase) family, improved VEGF-induced angiogenesis in vivo, as indicated by measurement of vascular density, and reduced vessel leakiness in an angiogenic model [124]. In addition, inhibition of Rac1 delayed healing in an experimental model of UC [121].

Thus, in contrast to VEGF, Rac1 seems to play a beneficial role in UC healing and may reverse VEGF effects in this disease. These studies indicate that the above angiogenic growth factors have different biologic features and actions, probably regulating different types of angiogenesis during mucosal healing in IBD.

Moreover, antiangiogenic factors are upregulated in the inflamed intestine, such as thrombospondin, endostatin—a cleaved fragment of collagen XVIII—and angiostatin—a cleaved fragment of plasminogen [28,125]. Thus, pathological angiogenesis or an increase in antiangiogenic signals, such as endostatin and angiostatin, might explain why mucosal lesions are resistant to repair in IBD.

The researchers at La Jolla University demonstrated in rat models of UC that an early increase in vascular permeability is initiated by histamine and exacerbated by upregulated VEGF [121]. Interestingly, elevated mucosal histamine levels are present in patients with UC, and increased levels of N-methylhistamine, a stable mast-cell metabolite, have been found in the urine of patients with active UC [126]. To determine whether colonic mast cells and increased release of histamine mediate the increased vascular permeability in the early stage of UC, these authors tested the effects of the mast cell stabilizer doxantrazole and histamine H1-receptor antagonists in the same experimental model of UC in rats. They found that blockade of mast cell secretion, as well as the H1-receptor, reduced increased vascular permeability and decreased signs of intestinal inflammation in rats [121]. These data provide a rationale for investigating cellular and molecular targets, such as histamine and mast cells, for the prevention and treatment of IBD, probably at an early stage. However, the microvascular changes that occur during human IBD have not been sufficiently investigated, and we are far from recognizing their involvement in the etiopathogenesis of the disease.

## 6. Conclusions

It has been suggested that, in IBD, intestinal vascular remodeling is disturbed by intestinal dysbiosis in the setting of intestinal inflammation. A defective immune response may promote intestinal angiogenesis through the selective induction of specific proangiogenic pathways. In turn, angiogenesis promotes the recruitment of immune cells via intestinal barrier dysfunction and sustains inflammation. However, an unresolved question in IBD pathogenesis is whether pathological angiogenesis is the cause or the consequence of intestinal inflammation mediated by the microbiota. Consequently, current antiangiogenic treatment strategies in IBD remain not fully satisfactory, and their effects probably depend on the phase of the disease. While intestinal microorganisms may activate the angiogenic cascade in IBD, modulation of the gut microbiome and other microbiota-targeted interventions may represent strategies for the prevention and/or treatment of pathological angiogenesis.

## Figures and Tables

**Table 1:** Principal proangiogenic components in IBD.

Principal Proangiogenic Components in IBD
Dysbiosis
Endothelial activation
Intestinal barrier dysfunction
Active inflammation
Hypercoagulability
Thrombus formation
Ischemia

**Table 2:** Manifestations of endothelial/vascular responses in IBD.

Endothelial activation and expression of adhesion molecules	Leucocyte recruitment
	Platelet adhesion
	Inflammation
VEGF expression	EC proliferation and migration
	Upregulation of adhesion molecules
	Immune-cell recruitment
	Vascular permeability
	Angiogenic sprouting
bFGF expression	Angiogenic sprouting
PDGF expression	Angiogenic sprouting
	Vascular coverage
Toll–like receptor expression by ECs	Regulation of endothelial-barrier homeostasis
	Specific receptor for bacterial products
Coagulation activation	Platelet adhesion and activation
	Impairment of the protein C pathway
	Thrombus formation
	Ischemia
Microvascular dysfunction	Granulomatous vasculitis
	Ulceration
Angiogenesis	Neovascularization
	Vascular remodeling
	Initiation/promotion of inflammation

Abbreviations: ECs: endothelial cells, VEGF: vascular endothelial growth factor, bFGF: basic fibroblast growth factor, PDGF: platelet-derived growth factor.

## Data Availability

No new data were created or analyzed in this study. Data sharing is not applicable to this article.
